# Oral health service utilization by elderly beneficiaries of the Mexican Institute of Social Security in México city

**DOI:** 10.1186/1472-6963-7-211

**Published:** 2007-12-21

**Authors:** Sergio Sánchez-García, Javier de la Fuente-Hernández, Teresa Juárez-Cedillo, José Manuel Ortega Mendoza, Hortensia Reyes-Morales, Fortino Solórzano-Santos, Carmen García-Peña

**Affiliations:** 1 Unidad de Investigación Epidemiológica y en Servicios de Salud, Área de Envejecimiento, Centro Médico Nacional Siglo XXI; Instituto Mexicano del Seguro Social, México, D.F., México; 2 División de Estudios Profesionales de la Facultad de Odontología, Universidad Nacional Autónoma de México, México, D.F., México; 3 Unidad de Medicina Familiar No. 28 Gabriel Mancera, Instituto Mexicano del Seguro Social, México, D.F., México; 4 Unidad de Investigación Epidemiológica y en Servicios de Salud, Centro Médico Nacional Siglo XXI; Instituto Mexicano del Seguro Social. México, D.F., México; 5 Departamento de Infectología, Hospital de Pediatría, Centro Médico Nacional Siglo XXI; Instituto Mexicano del Seguro Social. México, D.F., México

## Abstract

**Background:**

The aging population poses a challenge to Mexican health services. The aim of this study is to describe recent oral health services utilization and its association with socio-demographic characteristics and co-morbidity in Mexican Social Security beneficiaries 60 years and older.

**Methods:**

A sample of 700 individuals aged 60+ years was randomly chosen from the databases of the Mexican Institute of Social Security (IMSS). These participants resided in the southwest of Mexico City and made up the final sample of a cohort study for identifying risk factors for root caries in elderly patients. Sociodemographic variables, presence of cognitive decline, depression, morbidity, medication consumption, and utilization of as well as reasons for seeking oral health services within the past 12 months were collected through a questionnaire. Clinical oral assessments were carried out to determine coronal and root caries experience.

**Results:**

The sample consisted of 698 individuals aged 71.6 years on average, of whom 68.3% were women. 374 participants (53.6%) had made use of oral health services within the past 12 months. 81% of those who used oral health services sought private medical care, 12.8% sought social security services, and 6.2% public health services. 99.7% had experienced coronal caries and 44.0% root caries. Female sex (OR = 2.0), 6 years' schooling or less (OR = 1.4), and caries experience in more than 22 teeth (OR = 0.6) are factors associated with the utilization of these services.

**Conclusion:**

About half the elderly beneficiaries of social security have made use of oral health services within the past 12 months, and many of them have to use private services. Being a woman, having little schooling, and low caries experience are factors associated with the use of these services.

## Background

The increase in life expectancy of the population in general, particularly of the elderly all over the world, must be considered as a successful result of the progress of humankind. Progress in preventive and curative technologies for many illnesses, along with low exposure to risk conditions, should raise expectations of reaching old age in better health and living it adequately [1]. This ageing process poses a significant challenge to public health at a time when the persistence of poverty in countries that still face basic development problems, like Mexico, generates further pressure on their health systems, which are already overburdened [2].

One of the main components of care for this age group is oral health, especially in Latin America, where 75% of elderly people do not visit the dentist often enough and consequently suffer from very poor oral health, making it necessary to implement health policies that cover this unresolved need [3].

Literature on patterns of dental care services utilization has not been frequently reported. Apollonio et al [4] reported in a cross-sectional study covering 1,201 older people that an adequate dental status could be predicted by better educational and financial conditions. Randolph *et al.*[5] found in 3,050 Mexican-American non-institutionalized people aged 65 years and older that younger ages, more schooling and higher income were associated with recent visits to a dentist. This finding supports Anderson and Newman's original model, first developed in the seventies [6]. The purpose of this framework was to discover conditions that either facilitate or impede the utilization of oral care services. An individual's access to and use of health services is considered to be a function of three characteristics: 1) predisposing factors, i.e. the sociocultural characteristics of individuals that exist prior to their illness; 2) enabling factors, i.e. the logistical aspects involved in obtaining care; and 3) need factors, i.e. the most immediate cause of health service use. The framework was adapted for dental services by Kiyak [7] so as to include factors such as income and schooling. However, little is known about factors and patterns of dental care use in developing countries.

Data from the Mexican Institute of Social Security (IMSS) reveals that 87% of elderly women and 90% of elderly men regularly make use of medical services [8], but no information is known about dental care. Faced with a scenario of a growing ageing population, it is urgent to analyze their health needs in order to promote new schemes for the provision of health services and health policies.

Within this context, the aim of this study is to describe recent oral health services utilization and its association with socio-demographic characteristics and co-morbidity in Mexico City's IMSS beneficiaries aged 60 years or older.

## Methods

### Participants

The present study is a secondary analysis based on a sample consisting of 700 individuals randomly chosen from among 35,191 IMSS beneficiaries aged 60 or older, resident in the southwest of Mexico City, who made up the final sample of a cohort study for identifying risk factors for root caries in elderly people. Sample size for this study was re-calculated assuming a proportion of oral health services utilization of 20% and a confidence level of 95% (maximum precision of ± 3%). A sample of 682 participants was obtained. Every participant was visited at home as many times as necessary in order to decrease the possibility of losing participants. Institutionalized participants, those who refused to answer the questionnaire, and those unable to do so owing to severe cognitive impairment were excluded from the study.

### Setting

Mexico has a complex health organization. Public services for the uninsured population, usually poor, with an informal job, underemployed, or unemployed are provided by the Health Ministry. Social security services cover more than half the Mexican population; the IMSS (Mexican Institute of Social Security) covers private sector workers and the ISSSTE (Institute of Social Security and Services for State Workers) provides coverage for the civil service. There are other government bodies that provide their own workers with medical services, as is the case with PEMEX (Mexican Oil), the SEDENA (National Defense Department), and the SEDEMAR (Navy Department). The private sector's services are little-regulated; their users pay service providers their fees at market prices or through private medical insurance, which still has very limited coverage (3.0%) [9].

The IMSS offers health care and social security services to roughly half of the population and covers 64% of the elderly population in Mexico. It was created by law in 1943 and is funded by the government, employers, and employees. It is a social security system; therefore, the only requirement to be registered is to be employed, regardless of one's state of health. Workers, their parents, and other close relatives are assigned to a Family Medicine Unit, which is the primary health care provider. The IMSS offers a comprehensive package of benefits that include health care services at all levels of care and economic benefits such as a pension. Mexico City has a population of nearly 860,000 adults aged 60 years and older, 418,000 of whom (48.6%) are affiliated to the IMSS and thus constitute the population base for this study.

### Data collection

Data were collected after their mental state was evaluated and informed consent was obtained. Information was gathered through face to face interviews conducted by previously trained last-year dentistry students. It was not possible to include new variables because the present report is based on a secondary analysis.

Utilization of oral health services during the last twelve months was measured by direct questions inquiring about the type of service used, main reason for consultation, number of consultations, number and type of chronic diseases diagnosed by a physician (diabetes, hypertension, depression, cancer, Parkinson's disease, cardiac disease, osteoporosis, and arthritis), and number of drugs being taken. Cognitive impairment and depression were included because of their importance in old age. Both represent lack of functionality and are related to high rates of service use. The Mini-mental state examination (MMSE) was applied to evaluate cognitive state [10,11]. A score equal to or less than 23 points was positive for cognitive impairment. Depression was evaluated with the Geriatric Depression Scale -10 items [12,13]. A score equal to or greater than four points was positive for depression. Socio-demographic variables were age, gender, schooling and last qualification obtained, marital status, total income, and main occupation.

Clinical oral assessments were carried out to determine coronal and root caries experience. The clinical oral assessments were done by three dental surgeons who previously participated in a training and standardization course (Kappa > 0.85 inter- and intra-examiner) in accordance with the criteria recommended by the WHO to assess the state of dentition [14]. The assessment was done with the subject seated on a chair (in some cases in a wheelchair) under natural light, using a No. 5 mirror and a WHO-type periodontal probe (PCP 11.5B, Hu-Friedy).

The assessment of each tooth was done by observing the coronal and the root, starting with the second molar of the upper right quadrant, continuing with the upper left and lower left quadrants, and ending with the second molar of the lower right quadrant. When the subject had a removable prosthesis, it was taken out before beginning the clinical assessment.

From the clinical assessment, the coronal DMFT index (number of Decayed/Missing/Filled Teeth) and root surface DFT index (number of Decayed/Filled Roots), which represent present and past coronal and root caries experience, were calculated.

The 75 percentile in the coronal DMFT index and root DFT index was defined as a cut-off point to determine high coronal and root caries experience. The cut-off point defined for classifying high caries experience was coronal DMFT index > 22 teeth and/or root DFT index > two roots.

The interview and personalized clinical assessment were carried out in the participants' homes.

### Statistical methods

The analysis was done using SPSS (Statistical Package for the Social Sciences), version 10 for Windows. Bivariate analysis was performed to evaluate association among use of services, socio-demographic variables, and co-morbidity through the Chi^2 ^test and odd ratios. Logistic regression was used to derive adjusted estimates.

### Ethical approval

The IMSS' National Health Research Committee and Ethics Subcommittee of District No. 3 of the Southwest of the Federal District reviewed and approved the research protocol of which this study is a part (2002-721-0013).

## Results

Overall, 698 participants were interviewed and examined. The average age was 71.6 (± 1SD = 7.1). 68.3% of the participants were women, (age mean = 71.3, SD = 7.0) and 31.7% were men (age mean = 72.2, SD = 7.3). The frequency and distribution of the study variables are shown in Table 1.

**Table 1:** Sociodemographic charactheristics and diagnosis related to oral health services utilization.

	**Users**		**Non-users**		**Total**	
	n	%	n	%	n	%
**Sex**						
Female	279	74.6	198	61.1	477	68.3
Male	95	25.4	126	38.9	221	31.7
**Age (years)**						
60–74	252	67.4	209	64.5	461	66.0
75 or older	122	32.6	115	35.5	237	34.0
**Marital status**						
Single/divorced/widowed	202	54.0	154	47.5	356	51.0
Married	172	46.0	170	52.5	342	49.0
**Schooling**						
≤ 6 years	145	38.8	159	49.1	304	43.6
> 6 years	229	61.2	165	50.9	394	56.4
**Paid work**						
No	146	39.0	137	42.3	283	40.5
Yes	228	61.0	187	57.7	415	59.5
**Monthly income**						
< 4,000 MP*	168	73.7	149	79.7	317	76.4
≥ 4,000 MP	52	22.8	28	15.0	80	19.3
Not reported	8	3.5	10	5.3	18	4.3
**Cognitive decline**						
No	287	76.7	245	75.6	532	76.2
Yes	87	23.3	79	24.4	167	23.8
**Depression**						
No	243	65.0	197	60.8	440	63.0
Yes	131	35.0	127	39.2	258	37.0
**Morbidity**						
< 3 illnesses	276	73.8	234	72.2	510	73.1
≥ 3 illnesses	98	26.2	90	27.8	188	26.9
**Medication consumption**						
≤ 4	57	15.2	74	22.8	131	18.8
> 4	317	84.8	250	77.2	567	81.2
**Coronal DMFT**						
≤ 22	88	23.5	103	31.8	507	72.6
> 22	286	76.5	221	68.2	191	27.4
**Root DFT**						
≤ 2	264	70.6	210	64.8	474	67.9
> 2	110	29.4	114	35.2	224	32.1
**All combined**	374	53.6	324	46.4	698	100

*Mexican pesos

374 participants (53.6%) utilized oral health services within the past twelve months, 58.5% of the women and 43.0% of the men. 81% of the participants that made use of oral health services sought private care, 12.8% social security care, and 6.2% public care. The main reasons for oral care were dental examination, 44.4%; tooth restoration, 35.0%; dental cleaning, 32.1%; and one or more tooth extractions, 23.3%. Table 2 shows the distribution and frequency of oral health service utilization and type of health center.

**Table 2:** Reasons for seeking oral health services and type of health center visited by the elderly population.

	**Type of health center**			
	**Private n = 303**	**Social Security n = 48**	**Public n = 23**	**Total n = 374**
**Reasons for seeking care**	**%**	**%**	**%**	**%**
Dental examination	46.2	37.5	34.8	44.4
Tooth restoration	36.3	20.8	47.8	35.0
Dental cleaning	33.0	29.2	26.1	32.1
Tooth extraction(s)	18.8	47.9	30.4	23.3
Having a prosthesis made	23.7	6.3	26.1	21.7
Aching tooth or gums	19.1	27.1	21.7	20.3
Repairs to a prosthesis	13.2	8.4	0	11.7
Something else hurt	5.0	4.2	8.7	5.1
Endodontic treatment	4.3	4.2	0	4.0
Topical application of fluoride	1.7	2.1	0	1.6
Other reasons	1.3	0	0	1.1

The prevalence of coronal caries was 99.7%. The percentage was 99.8% in women and 99.5% in men. The average number of teeth with coronal caries was 2.4 (SD = 3.1). The average number of missing teeth was 12.2 (SD = 7.7). In the case of filled teeth, the mean was 2.6 (SD = 3.1). The average coronal DMFT index was 17.3 (SD = 6.1).

The prevalence of root surface caries was 44.0% (women = 40.7%, men = 51.1%). The average number of decayed roots was 1.2 (SD = 2.2) and that of filled roots was 0.1 (SD = 0.6). The average root DFT index was 1.4 (SD = 2.3).

Association between the variables of the study and the utilization of oral health services is shown in Table 3. There is an association between having more than six years' schooling and using oral health services within the past 12 months. High coronal caries experience is associated with lack of utilization of oral health services within the past 12 months. The associations mentioned above are statistically significant both for the raw and the adjusted analyses (p < 0.05). However, monthly income and oral care center were not included in Table 3 due to the fact that no significant association with use of services was found when the adjustment was performed.

**Table 3:** Strength of association between the utilization of oral health services and the variables under study.

**Variable**	**OR (95% CI)**	**p**	**Adjusted OR (95% CI)**	**p**
**Sex**				
Male	1		1	
Female	1.8 (1.3–2.5)	<0.001	2.0 (1.4–3.1)	<0.001
**Age**				
75 years or older	1		1	
60–74 years	0.8 (0.6–1.2)	0.409	0.9 (0.6–1.2)	0.618
**Marital Status**				
Single/divorced/widowed	1		1	
Married	0.7 (0.6–1.1.)	0.074	1.0 (0.7–1.4)	0.883
**Schooling**				
≤ 6 years	1		1	
> 6 years	1.5 (1.1–2.0)	0.008	1.4 (1.1–2.0)	0.020
**Paid Work**				
No	1		1	
Yes	1.1 (0.8–1.5)	0.376	1.3 (0.9–1.9)	0.114
**Cognitive Decline**				
No	1.0		1	
Yes	0.9 (0.6–1.3)	0.692	0.9 (0.6–1.3)	0.774
**Depression**				
No	1		1	
Yes	1.1 (0.8–1.2)	0.247	1.1 (0.8–1.6)	0.314
**Morbidity**				
≤ 3	1		1	
> 3	0.9 (0.6–1.2)	0.549	0.6 (0.3–0.9)	0.020
**Medication Consumption**				
≤ 4	1		1	
> 4	0.6 (0.4–0.9)	0.007	1.1 (0.7–1.6)	0.562
**Coronal DMFT**				
≤ 22	1		1	
> 22	0.6 (0.4–0.9)	0.015	0.6 (0.4–0.9)	0.024
**Root DFT**				
≤ 2	1		1	
> 2	0.7 (0.5–1.1)	0.104	0.8 (0.6–1.2)	0.487

OR: Odds ratio Adjusted

OR: Adjusted odds ratio among all the variables.

95% CI: 95% confidence interval

## Discussion

In summary, just over half of the participants had used oral health services, 81% of whom had to use private services. Female sex, higher schooling, and an experience of coronal caries in fewer than 22 teeth were associated with higher use of oral health services.

This study is important because it is the first one in the context of elderly beneficiaries of social security. However, we have to accept that it does not allow identifying predictive factors since the design was cross-sectional.

The elderly population has been advised to visit the dentist once a year minimum in order to benefit from preventive, diagnostic, control, and treatment services [15-17], as the effects in terms of oral health are positive [18]. Our results show that 53.6% of the elderly population has used oral health services within the past 12 months, a figure that is similar to that reported in other studies on the elderly population of industrialized countries [19,20]. This may be due to the fact that, even though elderly people are not able to determine their specific needs for treatment, they can opportunely refer themselves to an oral health specialist because their perception of their oral health is right [21]. However, the percentage of the elderly population that utilizes oral health services is very low compared to the percentage that uses medical services.

Considering the main reasons for oral care presented in this study, which were dental examination, tooth restoration, dental cleaning (prophylaxis), and tooth extraction; it is evident that the services provided by public health institutions, including the IMSS, do not satisfy the needs for care, which is why the elderly population must turn to private oral health services in order to fulfill them. At the IMSS and in the public system in general, oral health services are limited to prevention and control treatments, as well as restoration with amalgam or composite resin. All restoration and rehabilitation treatments must be covered by private oral health services, so, it may be possible that the old population with a lower income has even poorer oral health, compared with our participants.

In the study, participants were found with a high percentage of coronal and root caries. This finding reveals an important package of unresolved needs which includes oral restoration and oral rehabilitation [22-24], which are not covered by public institutions such as the IMSS because of their financial impact. However, a re-definition of health policies must be considered in the near future since the consequences of poor oral health are psychological, social, and also nutritional, and they could produce higher costs of care.

Regarding the utilization of oral health services and associated factors, women were twice as likely as men to have used oral health services. Reports from Europe and Canada [25,26] have found similar results. That pattern is also present when medical care utilization is analyzed; it is likely that illness perception and social roles could partly explain why women use oral health services more than men [27].

Likewise, various authors have asserted that schooling is related to regular use of oral health services [28-31]. In our study, we observed that there is an association between elderly people with more than six years' schooling and the utilization of oral health services, which is why we need oral health education programs for elderly people. However, it is important to take into account that higher oral services utilization could be the result of a higher income. Further research has to test this hypothesis.

One novel result is that people with more than 22 decayed teeth use oral health services less often, which coincides with another study that reports similar findings [32]. It has been reported that coronal caries experience (DMFT index) consists mostly of missing teeth in the elderly population, as shown by our results [33,34]. Natural tooth loss is one of the significantly negative variables that have an impact on health in general and on the quality of life of the elderly [35]. Besides, dental caries could be considered as an economic indicator of the significant expenditure on the elderly population since it increases the demand for oral health services [25]. Surprisingly, cognitive decline and the presence of depression were not associated with low rates of oral health services utilization.

The present study was based on the adapted Kiyak model of dental health services utilization [7], but it included only socio-demographic characteristics and the presence of oral diseases. Certainly, further research has to be done in order to integrate a predictive model for oral health services utilization. However, the underlying idea of the present study was to point out the relevance of the topic, since oral health programs in Mexico have been focused on children and dental problems in the elderly have been totally dismissed.

Finally, public health systems in countries like Mexico have to resolve the dilemma of oral care in the elderly. A healthy, independent ageing population with a high quality of life has to be achieved even with a scenario of scarce resources and financial strain.

## Conclusion

Based on the results of this study, around half the elderly beneficiaries of social security have made use of oral health services within the past 12 months, and many of them preferred to utilize private services. Less than six years' schooling, female sex, and high caries experience are factors that are associated with the utilization of these services.

## Competing interests

The author(s) declare that they have no competing interests.

## Authors' contributions

SS-G originated the idea for this study, did the research proposal, data analysis, and prepared the manuscript. JF-H, TJ-C, and JMO-M contributed to the research proposal, reviewed the analysis, and participated in the preparation of the manuscript. HR-M participated in the interpretation of the data and in the discussion of the paper. FS-S participated in the research proposal and reviewed the manuscript. CG-P designed and conducted the original proposal and was involved in the data analysis and in the preparation and discussion of the manuscript.

## Pre-publication history

The pre-publication history for this paper can be accessed here:


